# Exosomes derived from LPS-preconditioned bone marrow-derived MSC modulate macrophage plasticity to promote allograft survival via the NF-κB/NLRP3 signaling pathway

**DOI:** 10.1186/s12951-023-02087-8

**Published:** 2023-09-16

**Authors:** PeiYao Zhang, Panfeng Wu, Umar Zeb Khan, Zekun Zhou, Xinlei Sui, Cheng Li, Kangkang Dong, Yongjun Liu, Liming Qing, Juyu Tang

**Affiliations:** Department of Orthopedics, Hand & Microsurgery Surgery, Xiangya Hospital of Central South University, Xiangy Road, Changsha, 410008 Hunan China

**Keywords:** Mesenchymal stromal cells, LPS preconditioning, Exosome, Macrophage polarization, Allograft

## Abstract

**Objectives:**

This study investigated whether exosomes from LPS pretreated bone marrow mesenchymal stem cells (LPS pre-MSCs) could prolong skin graft survival.

**Methods:**

The exosomes were isolated from the supernatant of MSCs pretreated with LPS. LPS pre-Exo and rapamycin were injected via the tail vein into C57BL/6 mice allografted with BALB/c skin; graft survival was observed and evaluated. The accumulation and polarization of macrophages were examined by immunohistochemistry. The differentiation of macrophages in the spleen was analyzed by flow cytometry. For in vitro, an inflammatory model was established. Specifically, bone marrow-derived macrophages (BMDMs) were isolated and cultured with LPS (100 ng/ml) for 3 h, and were further treated with LPS pre-Exo for 24 h or 48 h. The molecular signaling pathway responsible for modulating inflammation was examined by Western blotting. The expressions of downstream inflammatory cytokines were determined by Elisa, and the polarization of macrophages was analyzed by flow cytometry.

**Results:**

LPS pre-Exo could better ablate inflammation compared to untreated MSC-derived exosomes (BM-Exo). These loaded factors inhibited the expressions of inflammatory factors via a negative feedback mechanism. In vivo, LPS pre-Exo significantly attenuated inflammatory infiltration, thus improving the survival of allogeneic skin graft. Flow cytometric analysis of BMDMs showed that LPS pre-Exo were involved in the regulation of macrophage polarization and immune homeostasis during inflammation. Further investigation revealed that the NF-κB/NLRP3/procaspase-1/IL-1β signaling pathway played a key role in LPS pre-Exo-mediated regulation of macrophage polarization. Inhibiting NF-κB in BMDMs could abolish the LPS-induced activation of inflammatory pathways and the polarization of M1 macrophages while increasing the proportion of M2 cells.

**Conclusion:**

LPS pre-Exo are able to switch the polarization of macrophages and enhance the resolution of inflammation. This type of exosomes provides an improved immunotherapeutic potential in prolonging graft survival.

**Supplementary Information:**

The online version contains supplementary material available at 10.1186/s12951-023-02087-8.

## Introduction

In the face of end-stage organ failure, organ transplantation has been recognized as one of the most effective treatments, but graft rejection raises a great challenge for long-term graft survival [1]. In transplantation biology, how to induce transplantation tolerance while avoiding long-term immunosuppression remains a key goal of research [2]. Recent articles have shown that macrophages are associated with allograft rejection [3]. More specifically, macrophages have been detected in acute antibody-mediated and cell-mediated rejection [4, 5], and have also been implicated in both mouse and human chronic allograft injury models [6, 7]. Some authors reported that macrophages accounted for up to 60% of immune cells in severe rejection after kidney transplantation. By examining the role of CD68^+^ CD206^+^ macrophages in kidney rejection after transplantation, Toki et al. [8] summarized that the number of macrophages was positively related to the expressions of whole-tissue inflammatory genes and negatively related to the allograft outcomes. These findings suggest that macrophages play a pivotal role in the allograft rejection process and that targeting macrophages is a promising strategy for improving immune tolerance and prolonging allograft survival.

With the growth of knowledge in the field of immunoregulation in recent decadea, cell-based therapy has emerged as a novel technique for attenuating transplant rejection [9]. Owing to advantages of good migratory ability, plasticity, paracrine activity, immune modulation, and regenerative properties, mesenchymal stem cells (MSCs) have been recognized as important candidates for cell-based therapeutics, especially in organ transplantation. Previous studies revealed that MSCs had strong anti-inflammatory effects and excellent immunomodulatory properties [10, 11]. Interestingly, the immunomodulatory and immunosuppressive functions of mesenchymal stem cells (MSCs) primarily rely on their paracrine effects [12]. Exosomes, which are composed of protein, lipid or microRNA cargos, are the primary paracrine effectors for MSCs [13–15]. Recent studies have shown that exposure to a lipopolysaccharide (LPS)-preconditioned culture environment can promote the functional properties and trophic effects of MSCs in defending inflammatory conditions [14, 16–18]. However, it remains uncertain whether exosomes from LPS-preconditioned MSCs (LPS pre-Exo) can induce allograft rejection by regulating macrophages.

The nuclear factor kappa B (NF-κΒ) pathway, as a critical proinflammatory pathway, contributes to M1 polarization[19]. Suppression of the NF-κΒ pathway has been demonstrated to deactivate the priming signal of the pyrin domain-containing 3 (NLRP3) inflammasome, leading to attenuation of the inflammatory cascade by promoting the transition from the M1 to M2 polarization state [20]. Many studies have indicated that NLRP3 inflammasome dysregulation is involved in several autoimmune and inflammatory diseases, as well as graft-versus-host disease (GvHD), and skin and corneal allograft rejection [21–25]. Nonetheless, the mechanism by which LPS pre-Exo mediates its immunomodulatory function through the NF-κΒ/NLRP3 pathway warrants further investigation.

In this study, we aimed to characterize the exosomes secreted by LPS-preconditioned bone marrow MSCs (LPS pre-BM-MSCs) so as to examine the effect of LPS pre-Exo on immunosuppressive allograft rejection and clarify the related mechanism. Our results revealed that LPS pre-Exo were able to effectively trigger macrophage polarization into the regenerative M2 phenotype and prolong the survival of skin graft. In addition, we also examined the NF-κB/NLRP3/ASC/procaspase-1 signaling pathway, which was shown to be involved in the therapeutic effect of LPS pre-Exo. Our research supported that LPS pre-Exo could prolong allograft survival as a therapeutic strategy.

## Results

### Characterization of LPS-pre-Exos

**Fig. 1:**
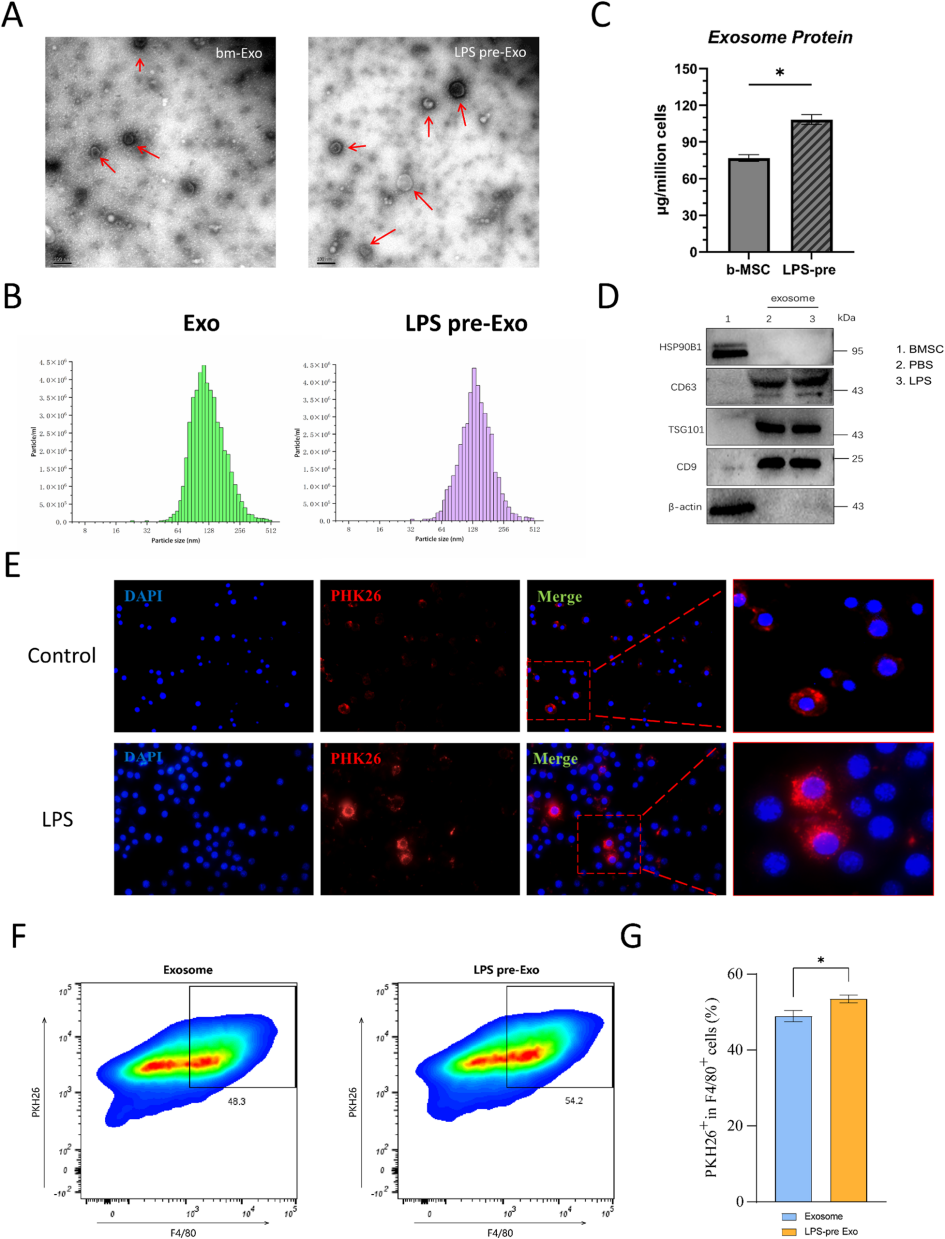
Identification of Exo. **A** TEM images of Exo and LPS pre-Exo. **B** The diameter of exosomes were detected by NTA analysis (n = 3). **C** The enhanced secretion of exosomes detected by BCA assay after LPS pretreatment (n = 3). Data are presented as mean ± SD.*P < 0.05. **D** The markers (HSP90B1, CD63, TSG101, CD9) of exosomes analyzed by Western blot. (Full-length blots are presented in Additional file 1: Fig. S2). **E** Macrophage phagocytosis of Exo and LPS Pre-Exo was observed under a fluorescence microscope. **F, G** Representative image of exosome (PKH26^+^) Uptake by Macrophages (F4/80.^+^) with flow cytometry analysis. Values are presented as the mean ± SD (n = 3). *P < 0.05

Bone marrow-derived MSCs were isolated from SD rats and preprocessed with 100 ng/ml LPS for 48 h. The BM-MSCs appeared to be irregularly triangular-shaped or spindle-shaped under microscope (Additional file 1: Fig. S1a). The MSC purity was examined by flow cytometry based on the positive marker expressions of CD29, CD44, and CD90 and the negative marker expression of CD45 (Additional file 1: Fig. S1c). The isolated BM-MSCs were found to be able to differentiate into osteoblasts, chondroblasts and adipocytes (Additional file 1: Fig. S1b). No significant apoptosis was observed in BM-MSCs treated with LPS after AMCYAN-Live staining (Additional file 1: Fig. S1d, e). The data confirmed that the original features of MSCs were not changed by LPS exposure. Then, exosomes were purified from the supernatant of BM-MSCs treated with or without LPS through gradient centrifugation and were verified by transmission electron microscopy (TEM), nanoparticle tracking analysis (NTA) and Western blotting, respectively. Morphologically, LPS-pre-Exos were cup‐shaped spherical vesicles in TEM images (Fig. 1A), which was not significantly different from BM-Exo. The specific markers of exosomes, CD63, CD9, Tsg101 and HSP90B1 (Fig. 1d), were detected by Western blotting, and no significant differences were shown between Exo and LPS pre-Exo. NTA analysis suggested that the size of Exo and LPS pre-Exo exhibited similar peaks around 128 nm (Fig. 1B). To evaluate the exosome release by MSCs and LPS-pre-MSCs, we also measured the protein concentration of each purified exosome by BCA assays. The results indicated that the total protein content of LPS pre-Exo was significantly elevated compared to the untreated group, implying that the MSC secretion of exosomes was enhanced by LPS stimulation for 48 h (Fig. 1C). We observed that PKH 26-stained exosomes were endocytosed successfully by BMDMs and localized around the nucleus (Fig. 1E). Subsequent flow cytometry analysis revealed that macrophages (F4/80^+^) exhibited higher efficiency in phagocytosis of LPS-pretreated exosomes (PKH26^+^) (Fig. 1F, G). These data showed that Exos and LPS-Exos were obtained successfully.

### LPS-pre-Exos significantly prolonged the survival time of skin grafts

**Fig. 2:**
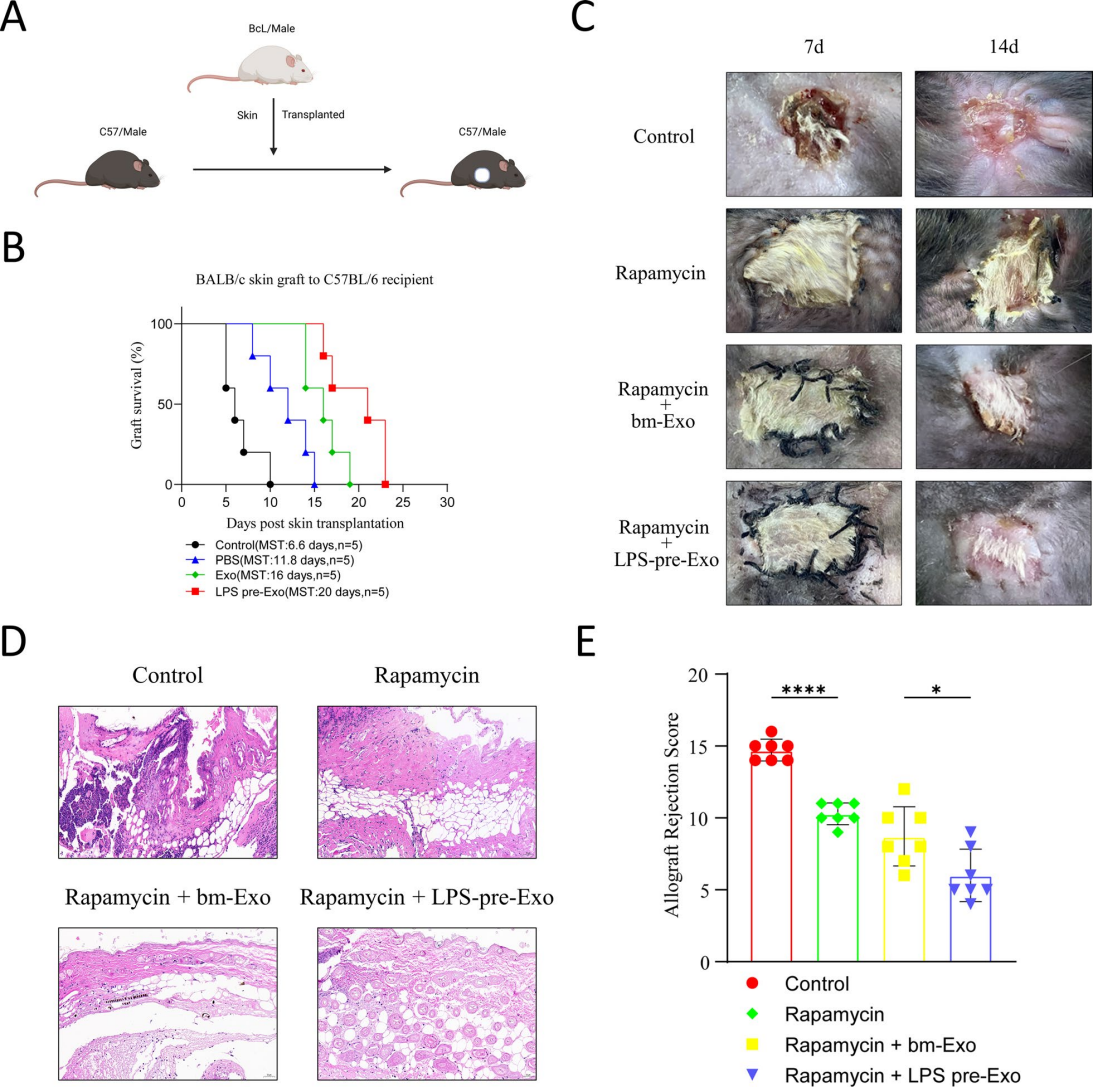
Effects of LPS pre-Exo on the survival of allogeneic skin grafts in vivo. **A** Schematic diagram of allogeneic skin grafting. The allografts Skin from BALB/c were transplanted into C57 recipient mice. **B**, **C** The necrosis of allograft skins was observed immediately after operation and at postoperative day 7, 14, 21, 23. Necrosis was observed at post-transplanted day 7 in the control group. The survival time of transplanted skins was prolonged by both rapamycin (1 mg/kg) and Exo (50 mg/kg), while LPS pre-Exo(50 mg/kg) was more effective (n = 5). **B** The percentage of allograft skin necrosis after transplantation (n = 5). **D**, **E** Representative H&E staining images of skin graft (day 7) in various groups. **E** Quantification of allograft rejection score in transplanted skin tissue in each group (n = 7). *P < 0.05, ****P < 0.0001

To clarify the effect of LPS-pre-Exos on allograft rejection, fully MHC-mismatched BALB/C skin allografts were transplanted into C57BL/6 mice which were treated with LPS-pre-Exos and the short‐term rapamycin‐based immunosuppressive therapy (Fig. 2A). The control mice were just treated with exosome-free PBS, and the remaining three groups were injected with rapamycin. The 3rd and 4th groups were additionally injected with Bm-Exos or LPS pre-Exos. Significant differences were found in allograft survival among the groups. Specifically, the Rapamycin group had a median survival time of 11.8 days, which was extended to 16 days with the addition of bm-Exos. Notably, the LPS pre-Exos group showed the longest median survival time of 20 days, significantly longer than the control group's median survival time of 6.6 days (Fig. 2B, C). All skin allografts in the control group were subjected to rejection on postoperative day (POD) 10. In addition, the survival time of skin grafts in the LPS-pre-Exo group was significantly longer than that in the other groups. The histopathology of graft skins on POD 7 was examined by H&E staining with allograft rejection scoring following the previously research methodologies [26] (Fig. 2D, E). The allografts from the controls exhibited histological signs of rejection and significant cellular infiltration, which was consistent with clinical outcomes. The skin samples collected from LPS-pre-Exo‐treated recipients showed no or minimal cellular infiltration, which supported that no or minimal histological signs of rejection were observed. These results implied that LPS pre-Exo could prolong the survival time of skin grafts.

### LPS-pre-Exos inhibited macrophage infiltration and attenuated immune rejection by switching the macrophage phenotype in vivo

**Fig. 3:**
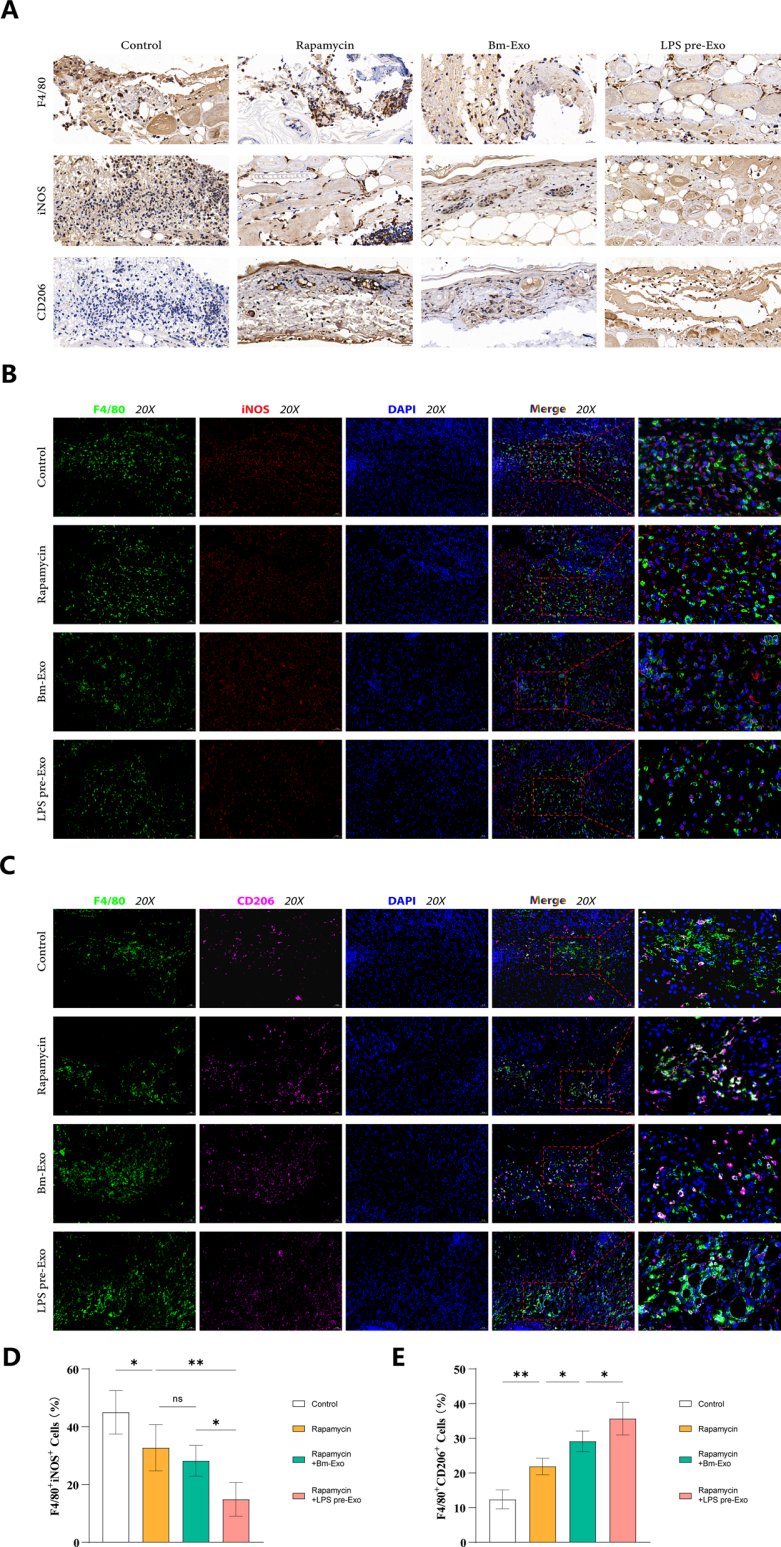
Effects of LPS pre-Exo on the polarization of macrophages in transplanted skin. The allogeneic skins were harvested at postoperative day 7. **A** Representative images of immunohistochemical staining of F4/80, iNOS and CD206 of transplanted skin in various groups. **B** Representative immunofluorescence images of F4/80 and iNOS in the transplanted skin tissues were obtained from each group of recipient mice. **C** Representative images of immunofluorescence of F4/80 and CD206 in allografted skin tissues of recipient mice in each group. **D**, **E** The data of positive cells in immunofluorescence images are shown as mean ± SD with One-way ANOVA analyses (n = 5). ns > 0.05, * P < 0.05, ** P < 0.005. F4/80: green; iNOS: red;CD206: magenta; DAPI: blue. Scale bars: 50 μm

**Fig. 4:**
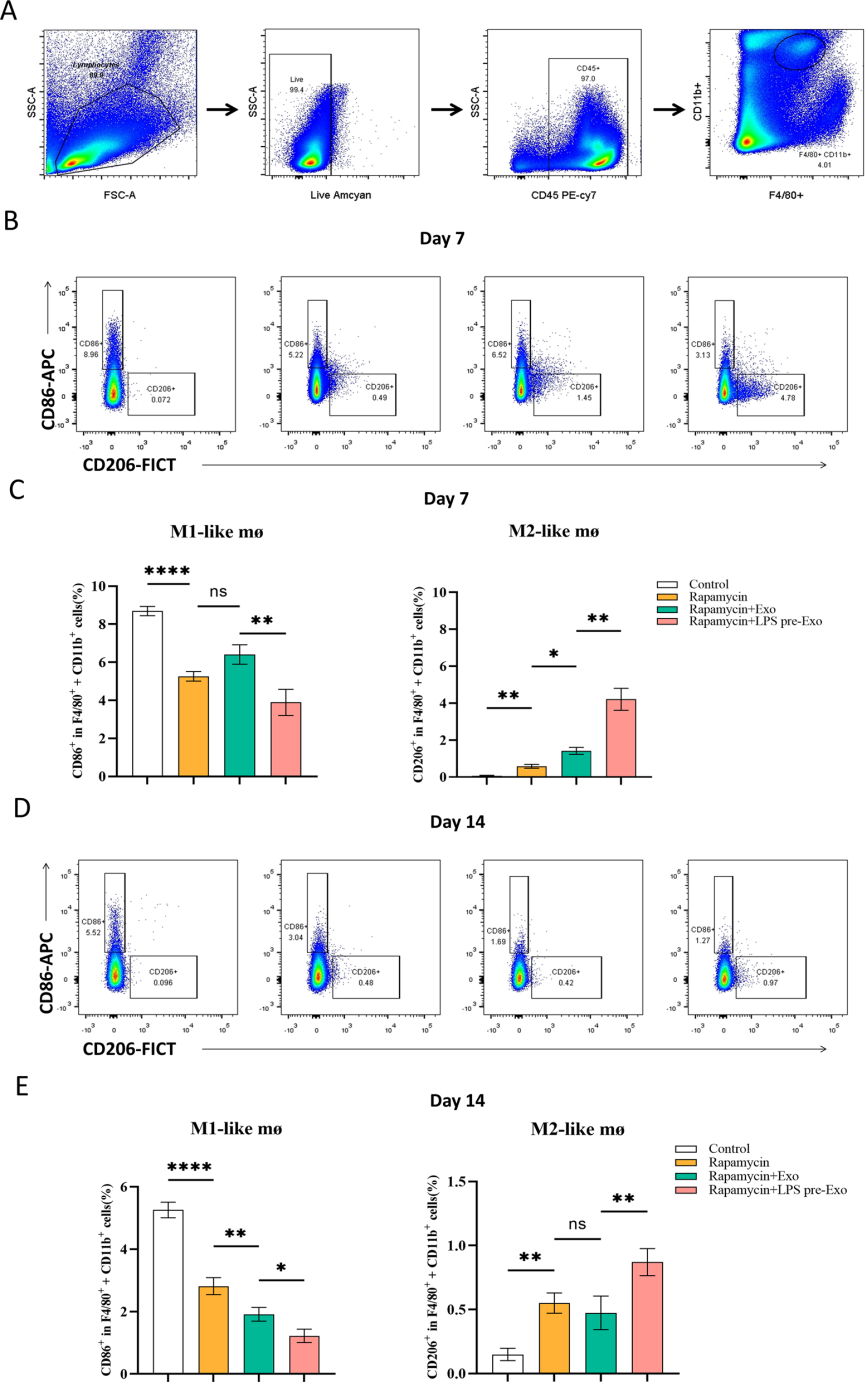
Effects of LPS pre-Exo on macrophage polarization in allograft recipients. **A** Gating strategy for detection of spleen macrophages by flow cytometry. **B**, **C** Representative images of CD86^+^and CD206^+^ at postoperative day 7 in various groups of spleen macrophages analyzed by flow cytometry. Values are presented as the mean ± SD (n = 3). ns > 0.05, *P < 0.05, **P < 0.005, **** P < 0.0001. **D**, **E** Representative images of CD86^+^and CD206.^+^ at postoperative day 14 in various groups of spleen macrophages analyzed by flow cytometry. Values are presented as the mean ± SD (n = 3). ns > 0.05, *P < 0.05, **P < 0.005, **** P < 0.0001

Macrophage polarization has been revealed to play a critical role in allograft rejection, and shifting the macrophage phenotype from M1 to M2 may attenuate immune response and prolong allograft survival. With this in mind, the effect of LPS pre-Exo on macrophage activity and phenotype of graft-infiltrating macrophages was investigated therapeutically in vivo. Specifically, the macrophage subpopulation was identified by immunohistochemical staining and immunofluorescence staining based on the molecular markers F4/80 (macrophage marker), iNOS (M1 marker) and CD206 (M2 marker). The results of our study showed that the addition of rapamycin led to a reduction in the expression of F4/80 and iNOS, while increasing the expression of CD206. Interestingly, we observed that exosomes had a synergistic effect with rapamycin, especially in the case of LPS pre-treated exosomes, leading to enhanced effects on CD206 expression (Fig. 3A). Similarly, in the immunofluorescence analysis, we found that exosomes also exhibited a synergistic regulation with rapamycin on F4/80 + iNOS + and F4/80 + CD206 + cell populations. Notably, the combination of LPS-pretreated exosomes with rapamycin had a more pronounced effect on these cell populations (Fig. 3B, C). To verify the effect of changes in macrophages subpopulation proportions on the immune tolerance in vivo, we further investigated the percentages of CD86^+^ and CD206^+^ cells on POD 7 and 14 in the blood of recipient mice. On POD 7, the administration of rapamycin resulted in a significant reduction in M1-like macrophages, and this effect was further enhanced when combined with LPS pre-treated exosomes. Regarding M2-like macrophages, all exosomes augmented the promotive effect of rapamycin, but LPS pre-treated exosomes demonstrated a stronger effect (Fig. 4A–C) (n = 3). Moving to POD 14, the number of F4/80^+^CD86^+^ and F4/80^+^CD206^+^ cells showed a similar trend as observed on POD 7. However, an interesting observation was made as time passed. The inhibitory effect of control exosomes on F4/80^+^CD86^+^ macrophages polarization increased, while no significant difference was observed in F4/80^+^CD206^+^ cells number compared to the rapamycin group (Fig. 4D, E) (n = 3). The combined evidence demonstrates that LPS pre-Exo is able to inhibit M1-like macrophage infiltration and facilitate M2 macrophage polarization.

### LPS pre-Exo inhibited the proliferation of primed macrophages and induced M2 macrophage polarization in vitro

**Fig. 5:**
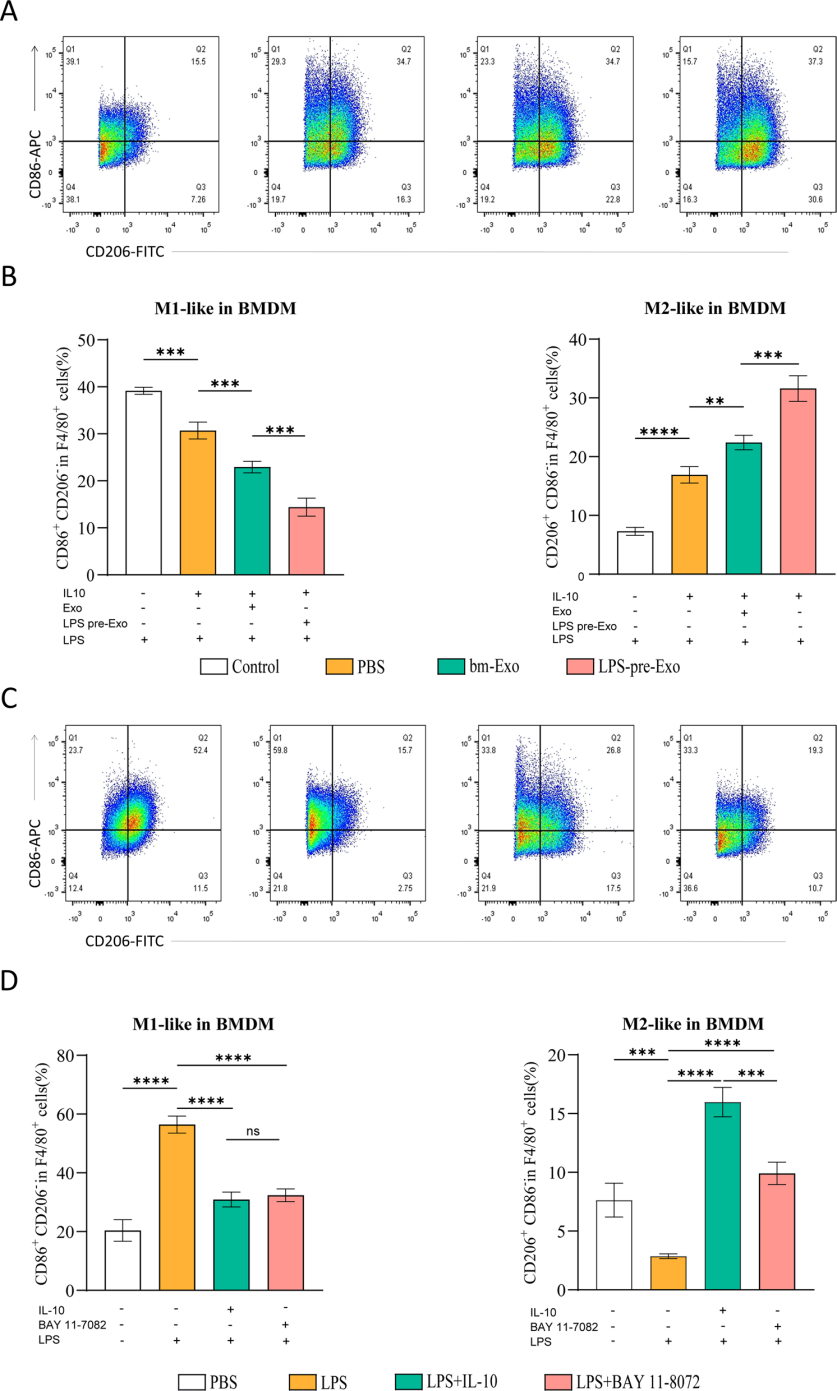
Effects of LPS pre-Exo on macrophage polarization in vitro. **A**, **B** BMDM cells were stimulated with LPS for 3 h, followed by Exo or LPS pre-Exo treatment for 24 h. IL-10 were added to the experimental groups for 24 h(n = 3). **C**, **D** The effect of the NF-κB inhibitor, BAY 11–8072 on the polarization of macrophages. **B**, **D** Representative images of CD86^+^ and CD206.^+^in different groups of BMDM cells analyzed by flow cytometry. Values are presented as the mean ± SD (n = 3). ns > 0.05, **P < 0.005, *** P < 0.0005, **** P < 0.0001

**Fig. 6:**
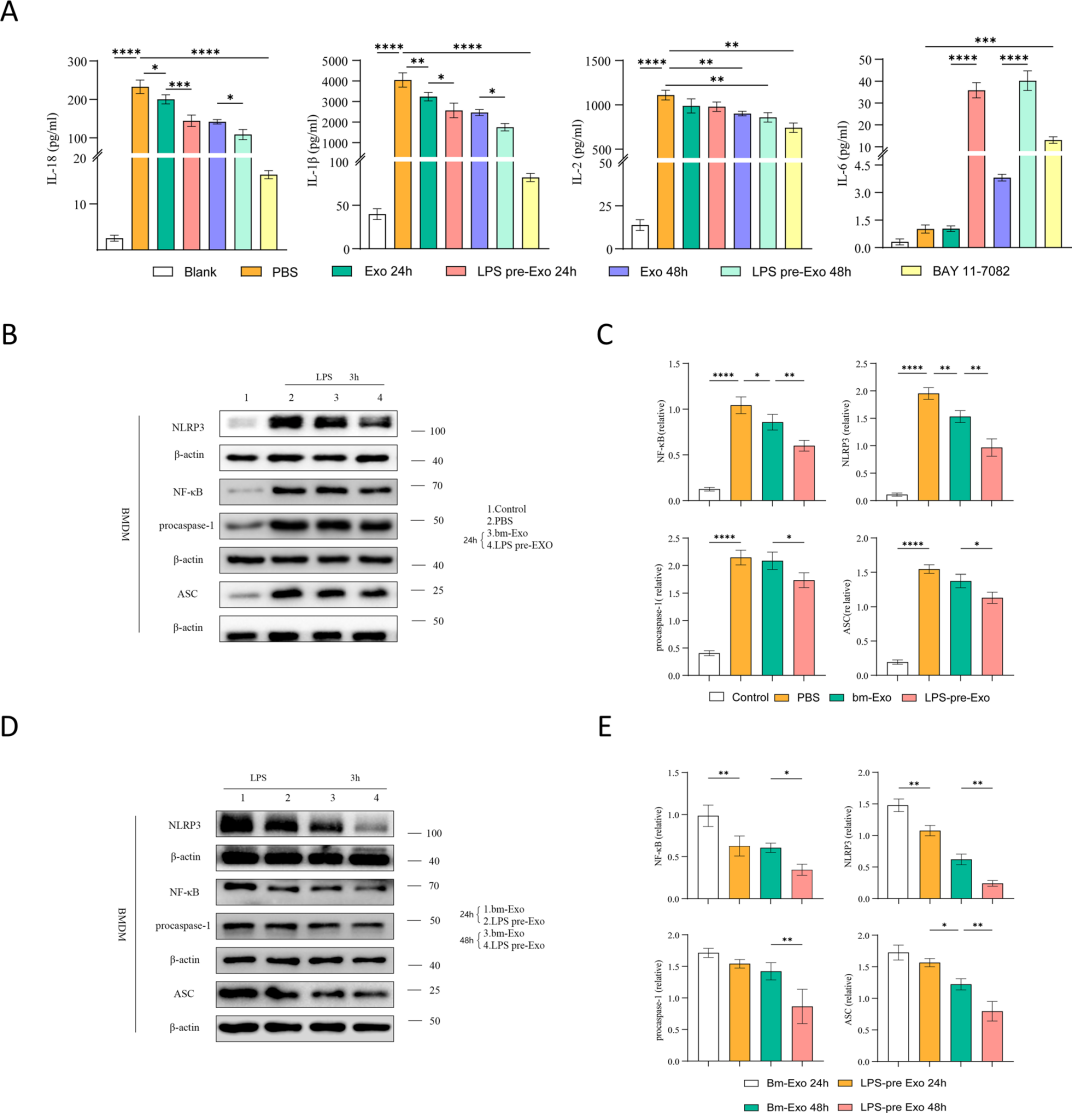
Effects of LPS pre-Exo on protein expression levels in macrophages. **A** The concentrations of cytokine (IL-2, IL-6, IL-1β, IL-18) were detected by Elisa in the macrophage supernatant of each group.(n = 4). **B**, **C** BMDM cells were pretreated with LPS (100 ng/m) for 3 h before culturing with LPS pre-Exo for 24 h. Protein expressions of NF-κB, NLRP3, Procaspase-1 and ASC in macrophages of each group analyzed by western blot. (Full-length blots are presented in Additional file 1: Fig. S3) The effect of LPS pre-Exo on protein expression in the macrophages compared with bm-Exo (n = 3). **D**, **E** BMDM cells were pretreated with LPS (100 ng/m) for 3 h before culturing with LPS pre-Exo for 48 h. Protein expression of NF-κB, NLRP3, Procaspase-1 and ASC in the macrophages of each group analyzed by Western blot. (Full-length blots are presented in Additional file 1: Fig. S4) The effect of LPS pre-Exo on protein expression in the macrophages compared with bm-Exo. (n = 3). **A**, **C**, **E** Data are presented as mean ± SD. *P < 0.05, **P < 0.005, *** P < 0.0005, **** P < 0.0001

To examine whether LPS pre-Exo could inhibit the proliferation of primed macrophages and accelerate the polarization of M2 macrophages in vitro, after labeling LPS pre-Exo with a red fluorescent dye (PKH26), they were co-cultured with macrophages to observe whether the LPS pre-Exo would be internalized by BMDMs or not. The results of fluorescence microscopy confirmed the internalization of the labelled exosomes by macrophages (Fig. 1E). Next, to determine whether LPS pre-Exo could induce the shifting from proinflammatory macrophages (M1) to anti-inflammatory macrophages (M2) in vitro, the M1 macrophage marker CD86 and the M2 macrophage marker CD206 were used respectively to identify macrophage subpopulations through flow cytometry. The results showed a significant increase in the density and distribution of M2 macrophages, and a significant reduction in those of M1 macrophages in the LPS pre-Exo group. Similar results were obtained with the NF-κB inhibitor BAY 11–8072 (Fig. 5A–D). M2 macrophages have been found to secrete cytokines (IL-6), while proinflammatory cytokines (IL-1β, IL-18, IL-2) have been associated with the polarization of M1 macrophages [19, 27, 28]. We then performed ELISA to further detect the relative expression levels of proinflammatory cytokines (IL-1β, IL-18, IL-2) and cytokines (IL-6), and the results indicated that BMDMs produced significantly more IL-6 and less IL-1β, IL-18, and IL-2 at 48 h in the LPS pre-Exo group (Fig. 6A). The combined evidence showed that LPS pre-Exo induced an immunosuppressive phenotype in activated macrophages and effectively inhibited the release of inflammatory cytokines. These findings indicate that LPS pre-Exo have a great therapeutic potential for improving allograft survival by boosting the polarization of M2 macrophages while inhibiting the proliferation of primed macrophages.

### LPS pre-Exo regulated macrophage activity via NF-κB/NLRP3/ASC/Procaspase-1 signaling

Previous studies have shown that LPS pre-Exo can induce the polarization of anti-inflammatory M2 in macrophages. To examine the molecular pathways that are targeted and affected by LPS pre-Exo in suppressing acute rejection in skin allografts, we investigated the expression of NLRP3 and the related proteins in BMDMs (Fig. 6B). Compared to the control group, LPS stimulation for 3 h led to a significant increase in the protein expression of NLRP3, NF-κB, procaspase-1, and ASC. However, after the addition of normal exosomes for 24 h, the stimulatory effect of LPS on NLRP3 and NF-κB expression was significantly inhibited, while the expression levels of procaspase-1 and ASC showed no significant changes. After treatment with LPS pre-Exos for 24 h, the protein expression levels of NLRP3, NF-κB, procaspase-1, and ASC were significantly downregulated compared to both the PBS group and the Bm-Exo group (Fig. 6C). The time-dependent effect of exosome action was explored by comparing the protein expression levels after co-culturing for 24 h and 48 h (Fig. 6D). The results revealed that after co-culturing for 24 h, the inhibitory effect of LPS pre-Exos on NLRP3 and NF-κB expression was stronger compared to control exosomes. The protein expression levels of NLRP3, NF-κB, and procaspase-1 in BMDM cells treated with LPS pre-Exos for 24 h were not significantly different from those treated with Bm-Exos for 48 h, except for ASC expression, where the inhibitory effect of Bm-Exos at 48 h was better than that of LPS pre-Exos at 24 h.Furthermore, after co-culturing for 48 h, the inhibitory effect of LPS pre-Exos on the protein expression levels of NLRP3, NF-κB, procaspase-1, and ASC was more pronounced compared to control exosomes (Fig. 6E). The results revealed that LPS pre-Exo inhibited NLRP3, ASC, procaspase-1 and NF-κB activation in BMDMs (Fig. 6C), and the expression levels of inflammatory proteins in BMDMs were also significantly downregulated 48 h after treatment (Fig. 6E).

### The miRNA profile of Bm-Exos was significantly altered by LPS

**Fig. 7:**
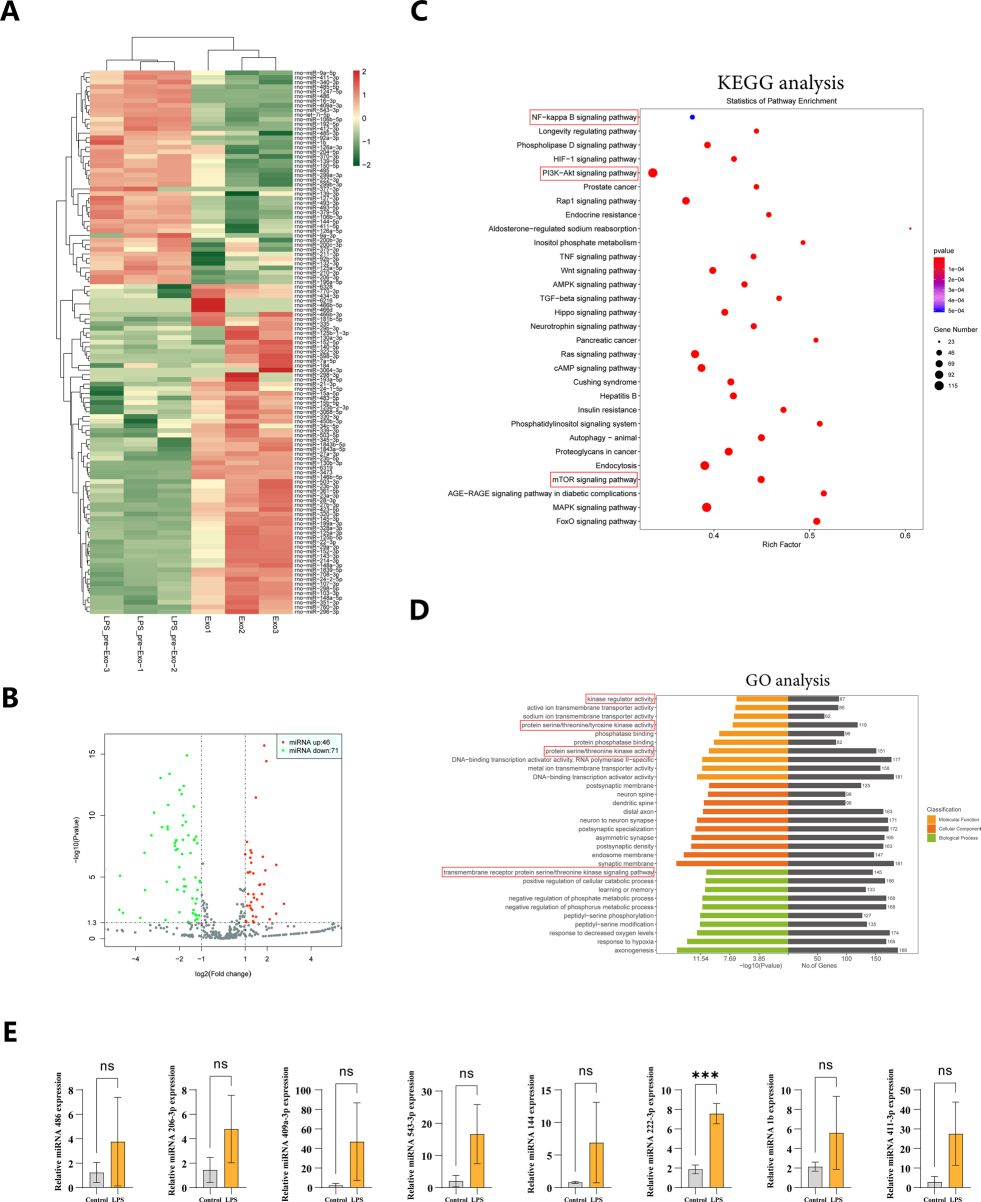
The expression of MiRNA-222-3p in LPS pre-Exos was sharply increased and it can regulate macrophages through exosomal transfer. **A** The heatmap showed the differential expression of miRNAs between Bm-Exo group and LPS pre-Exo group (n = 3). (Fold change > 1.0; *P* < 0.05). **B** The volcano plot demonstrated the altered expression levels of miRNAs between Bm-Exos and LPS pre-Exos. **C** The KEGG analysis of target genes associated with differentially expressed miRNAs showed that 30 signaling pathways were significant enriched (*P* < 0.05). **D** Gene Ontology (GO) analysis was conducted on the target genes of differentially expressed miRNAs (*P* < 0.05). **E** The qPCR analysis demonstrated the differential expression level of miR-486, miR-206-3p, miR-409a-3p, miR-543-3p, miR-144, miR-222-3p, miR-1b, and miR-411-3p in Bm-Exo group and LPS pre-Exo group. Data are presented as mean ± SD (n = 3). *** P < 0.0005

**Fig. 8:**
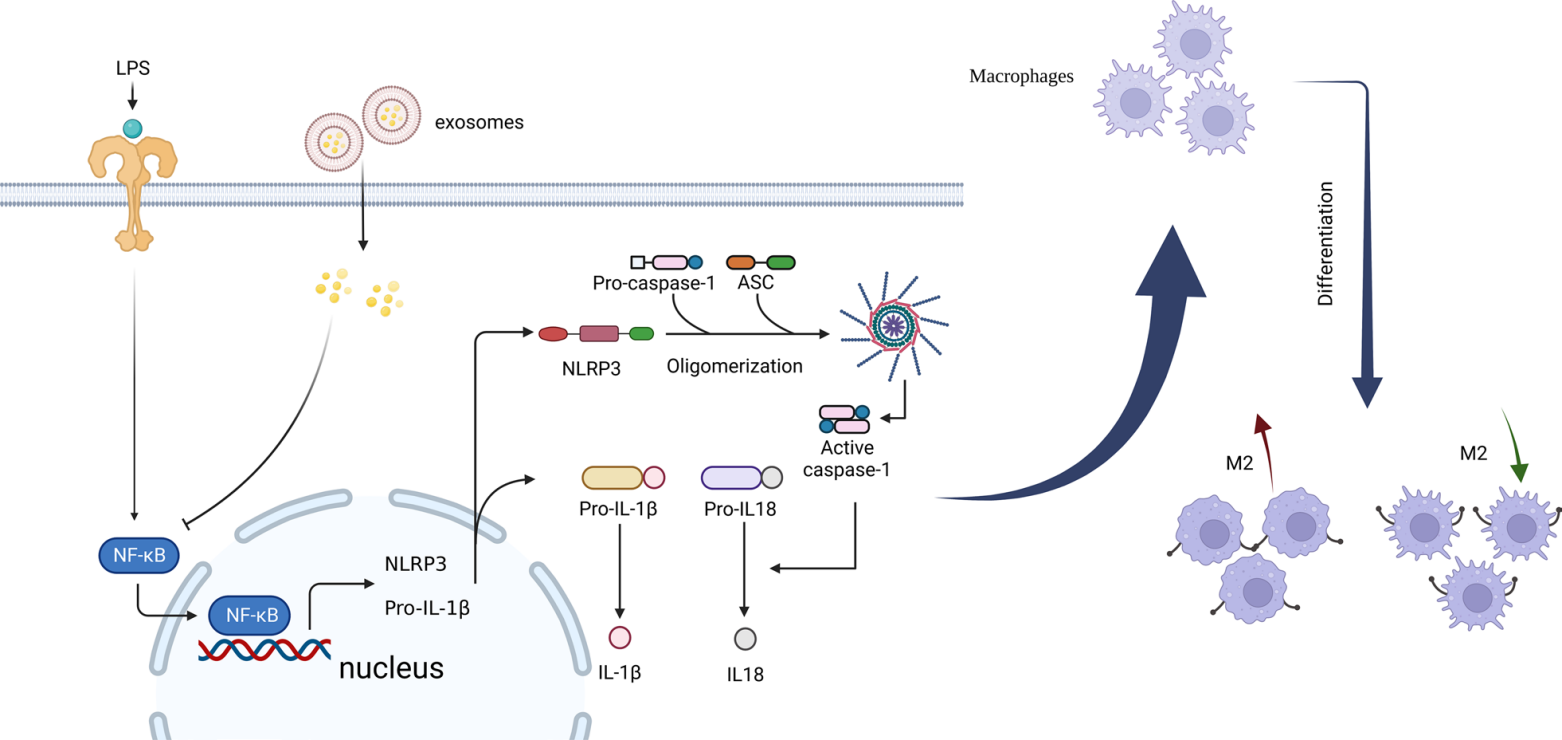
The simulation of signaling pathway by which Exo/LPS pre-Exo modulates the immune response to allografts. **Step 1**: LPS was recognized by macrophages and induced upregulation of NLRP3, IL-1β and IL-18 expression through the NF-κB signaling pathway. **Step 2**: After being endocytosed, LPS pre-Exo interacted with the NF-κB for preventing its activation and inhibited the expression of NLRP3 with suppressing the synthesis of NLRP3 inflammasome and subsequent production of IL-1β and IL-18. **Step 3**: In the subsequent immune response, following administration of LPS pre-Exo which induced alternation in the polarization of macrophages for expanding the ratio of M2 with CD86^+^ macrophages. Taken together, the functional outcome is to suppress the innate immune response

Exosomes transport various signaling molecules in intercellular communication, miRNA has been identified as a crucial player. For further insights into the underlying mechanisms of the aforementioned effects induced by LPS pre-Exos, we performed miRNA sequencing (Fig. 7A). The results demonstrated that LPS pre-Exos exhibited an upregulation in the expression of 46 miRNAs with 71 miRNAs showed a downregulation, in comparison to Bm-Exo (Fig. 7B). KEGG analysis revealed that target miRNAs were enriched in NF-κB signaling pathway, PI3K-Akt signaling pathway, autophagy and mTOR signaling pathway, which are classical siganal in inflammatory response and immune regulation (Fig. 7C). Further GO analysis revealed that the analyzed target miRNAs could affect biological processes from modulating molecular functions, including transmembrane receptor protein serine/threonine kinase signaling pathway, positive regulation of cellular catabolic process, negative regulation of phosphate metabolic process, response to hypoxia, and exert their regulatory effects on molecular functions through kinase regulator activity, protein serine/threonine/tyrosine kinase activity, protein serine/threonine kinase activity, DNA-binding transcription activator activity (specific to RNA polymerase II), and DNA-binding transcription activator activity (Fig. 7D). Following the criteria (P < 0.001, |log2(fold change)|> 1.8), we selected 8 significantly upregulated miRNAs, including miR-486, miR-206-3p, miR-409a-3p, miR-543-3p, miR-144, miR-222-3p, miR-1b, and miR-411-3p. Subsequently, we examined their corresponding expression levels and found a significant increase in the expression of miR-222-3p loaded by LPS pre-Exos compared to normal exosomes (Fig. 7E). Collectively, these data demonstrated that LPS pre-Exo prolonged allograft survival by inhibiting the proliferation of M1 macrophages, which was dependent on the attenuation of NF-κB/NLRP3 pathway by miRNA-222-3p (Fig. 8).

## Discussion

As small vesicles secreted by cells, exosomes can be loaded with a variety of cellular biological factors for intercellular communication [29, 30]. Some studies have shown that the upregulation of miRNA-222-3p could induce immune escape [31, 32]. Previously, it has been reported that MSC-derived exosomes exhibited a notable therapeutic effect on lung injury by inhibiting NF-κB activation or suppressing the release of inflammatory factors, such as IL-1β, IL-6 and TNF-α [33–35]. Meanwhile, NF-κB and the NLRP3 inflammasome were found to activate the polarization of antigen-presenting cells to further cause allograft rejection [36, 37]. Obviously, the factors loaded into exosomes were affected by the cell state [38, 39]. The superior inhibitory effect of exosomes derived from LPS-pretreated MSCs (LPS pre-Exo) has already been demonstrated [14, 40]. In the current study, we aimed to delineate the specific effect of LPS pre-Exo on allograft survival and to elucidate the corresponding mechanism of LPS pre-Exo in inducing immune tolerance. LPS pre-Exo were previously examined in the allogeneic skin graft model of mice [41]. Our findings suggested that LPS pre-Exo could significantly prolong survival (MST = 20 days) compared to the control treatment (MST = 6.6 days; Fig. 2). In earlier studies, no obvious tumorigenicity or immune rejection was observed after using exosomes [42, 43], but the safety and cytotoxicity of LPS pre-Exo still require further investigation [40, 43]. Considering the immunosuppressive effect and half-life of exosomes, we chose a dose of 50 mg/kg for the mouse model to decipher the potential inhibitory mechanism [44].

The NLRP3 inflammasome, which is an intracellular multimeric protein signaling complex, can activate potent inflammatory responses and has been associated with many inflammatory and autoimmune diseases, including diabetes, atherosclerosis, asthma, systemic lupus erythematosus and rheumatoid arthritis [22, 23, 45]. NLRP3 plays a pivotal role in the activation and assembly of NLRP3 inflammasome [46]. It can respond to pathogens (PAMPs) and endogenous activators (DAMPs), such as microbes, extracellular ATP, viral RNA, mitochondrial dysfunction, endoplasmic reticulum (ER) stress, and lysosomal rupture [47–50]. Activated inflammasome complexes contribute to the proliferation and differentiation of proinflammatory macrophages as well as tissue damage by mediating the activation of caspase-1 and the secretion of proinflammatory cytokines (IL-1β and IL-18) [51, 52]. Shi et al. reported that caspase-1 cleavage and the activation of NLRP3 inflammasome promoted the polarization of M1 macrophages so as to exacerbate liver damage [53]. Wu et al. found that in GvHD models, activated NLRP3 inflammasomes induced the polarization of M1 macrophage, which resulted in Th1 and Th17 differentiation and exacerbated the disease [21]. Moreover, allograft survival was affected by macrophage polarization via the NLRP3 inflammasome. Tian et al. showed that M1 polarization was reduced by inhibiting the postoperative activation of the NLRP3/IL-1β axis, thereby alleviating rejection in a rat corneal transplant model [54]. Amores-Iniesta et al. suggested that the P2X7 receptor in macrophages could recognize the extracellular ATP released by donor immune cells to activate the NLRP3 inflammasome and promote the maturation of IL-18 to signal immune rejection in mouse skin graft models [25]. Zhu et al. used melatonin to inhibit the activation of NLRP3 inflammasome and reduce the expression of IL-1β in mouse corneal transplant models, which decreased the infiltration of macrophages into the graft and improved the survival rate [24]. Zou et al. indicated that Pcsk9 depletion suppressed the macrophage recruitment and reduced vascular injury in mice with abdominal aorta allografts, which was associated with the blocking of NLRP3 inflammasome activation [55]. Furthermore, LPS-Exos have also been found to affect the NF-κB or NLRP3 signaling pathway, regulate macrophage polarization, reduce inflammation and facilitate tissue repair in kidney injury, diabetes, nerve injury, and myocardial infarction disease models [14, 34, 40, 56]. Du et al. revealed that the NF-κB signaling pathway was activated by DAMPs generated after allogeneic transplantation that were recognized by TLR4 on antigen-presenting cells via the classical pathway and initiated synthesis of the NLRP3 inflammasome [37]. Therefore, it was speculated that LPS pre-Exo could exert an immunosuppressive effect on the mouse skin allograft by down-regulating the NLRP3 inflammasome pathway.

On POD 7, we observed that LPS pre-Exo reduced the infiltration of F4/80^+^ and CD86^+^ macrophages and increased the percentage of CD206^+^ macrophages in allogeneic skin grafts. Moreover, a similar change in macrophage polarization was revealed in the spleen cells of recipient C57 mice (Figs. 2, 3, 4). In this study, the effect of LPS pre-Exo on macrophage polarization was examined by pretreating BMDMs with LPS. BMDMs were found to inhibit the differentiation of CD86^+^ macrophages and increase the proportion of CD206^+^ macrophages more effectively after treatment with LPS pre-Exo (Fig. 5). Further, LPS pre-Exo attenuated the LPS-induced activation of NF-κB, inhibited the expressions of NLRP3, procaspase-1, ASC, IL-1β, IL-18 and IL-2, and promoted the expression of IL-6 (Fig. 6). This finding contrasted with some previous findings [34, 57]. Interestingly, several reports indicate a close relationship between the polarization of M2-like macrophages and elevated expression levels of IL-6 [58–60]. This phenomenon might be attributed to the complex and diverse regulatory mechanisms mediated by IL-6 in macrophages [61, 62]. Further miRNA sequencing and Real-time RT-PCR analysis revealed that miRNA-222-3p may play a key role in the observed experimental results. The above results showed that miRNA-222-3p loaded by LPS pre-Exo prolonged the allograft survival time by inhibiting NF-κB and exerting an immunosuppressive effect against acute rejection by down-regulating NLRP3 expression. After allogeneic transplantation, alloantigens, including LPS and DAMPs, were significantly increased in recipients and were recognized by TLR4 in macrophages, which initiated the classical inflammatory cascade through the NF-κB signaling pathway to induce the expressions of NLRP3, ASC, IL-1β and IL-18. Upon being endocytosed, LPS pre-Exo could prevent the activation of NLRP3 inflammasome by interacting with NF-κB via miRNA-222-3p and inhibiting NLRP3 expression, thus preventing the activation of IL-1β and IL-18 in recipient macrophages. Subsequently, the macrophage polarization to CD86^+^ cells was decreased by LPS pre-Exo, the proportion of CD206^+^ macrophages and the release of IL-6 were increased, while the IL-2 secretion was reduced. Overall, the innate immune responses were suppressed. In this paper, we mainly focused on the regulatory effect of LPS pre-Exo on macrophage polarization after allograft transplantation, and the in vitro results of NLRP3 and the related molecules were used to elucidate the potential role of LPS pre-Exo.

We investigated the expressions of NF-κB and the related molecules in BMDMs after LPS pre-Exo treatment in vitro but did not examine the differentiation of extracellular ATP, P2X7 receptor, or Treg or Th17. Therefore, the results could not be comprehensively integrated. In addition, we only performed molecular and histological studies on the data 7 days after transplantation (acute rejection). However, due to a lack of data with longer time points, we were unable to confirm whether the immune status of recipients after LPS pre-Exo treatment, the persistence of CD86 + macrophages, and the downregulation of NLRP3 and its related molecules should be regarded as chronic rejection, delayed acute rejection, or real transplant tolerance. Thus, our results were still inadequate. Moreover, due to the absence of necessary resources, this study only examined the changes in miRNAs loaded in LPS pre-Exo without their associated molecular mechanisms, which shall be explored in the future. Admittedly, further experiments need to be conducted to address the above issues and to clarify the differences in miRNAs loaded in LPS pre-Exo and the underlying molecular mechanisms.

Our study demonstrates that the exosomes with miRNA-222-3p secreted from MSCs after LPS treatment may have better alleviation and regulation capabilities to balance macrophages and suppress inflammatory responses. LPS pre-Exo possessed a visible advantage in shifting macrophages from a proinflammatory M1 phenotype to an immunosuppressive M2 phenotype to prolong allotransplantation survival. The changes in macrophage polarization suggests that transferring exosomes released by preconditioned MSCs may have a significant therapeutic potential in the future treatment of allografts.

## Conclusions

The results of this study demonstrate that LPS pre-Exo can inhibit M1 polarization and promote M2 proliferation. Furthermore, LPS pre-Exo loaded with miRNA-222-3p target the NF-κB pathway and inhibit the expressions of NLRP3/caspase-1/IL-1β to promote inflammation reduction. In allogeneic murine models, LPS pre-Exo were found to switch the phenotype of infiltrated macrophage and induce immune tolerance to prolong skin graft survival. Overall, modified MSC-Exos can serve as the basis for future immunosuppressive therapy to prolong allograft survival.

## Method

### Cell preparation and in vitro treatment

Primary bone marrow-derived MSCs (Bm-MSCs) were isolated from three-week-old SD rats following the procedure previously reported [63]. The isolated cells were cultured in the minimum essential medium-alpha (MEM-α; Gibco) containing 10% exosome-free fetal bovine serum (FBS; Gibco). The cultures were maintained under the conditions of 37 °C, 95% humidity and 5% CO_2_, and the 3rd–4th passage cells were collected for experimental purposes. To verify the purity of isolated BM-MSCs, purified cells were stained for specific markers and verified as CD45^−^, CD29^+^, CD44^+^, and CD90^+^ by flow cytometry.

Preconditioning with LPS was performed once the cells had reached 70–80% confluence. After aspirating the medium and rinsing it with the phosphate buffered solution (PBS) for three times, the experimental cells were treated by LPS (100 ng/ml, Sigma), while the negative control was treated by PBS [38]. After resting for 48 h, the supernatant was taken to perform follow-up experiments.

Primary bone marrow-derived macrophages (BMDMs) were extracted from C57BL/6 J mice following the procedure described previously. The BMDMs were cultured in low-glucose Dulbecco's modified Eagle’s medium (DMEM) (Gibco) containing 10% FBS, 1% penicillin/streptomycin and M-CSF (10 μg/ml, Sigma). The medium was replaced once in 2 days, and experiments were performed on the 5th day of differentiation [64].

### Purification of exosomes

To purify the exosomes, cells were cultured following the procedure described in previous studies [65, 66]. After filtering with a 0.22-µm filter, the culture supernatants were centrifuged at 3,000 g at 4 °C for 30 min (Thermo Fisher) first, and then further centrifuged at 10,000 g at 4 °C for 1 h (Beckman Coulter, Optima XPN-100) to remove cell debris, dead cells and large vesicles. Subsequently, after centrifugation at 100,000 g at 4 °C for 2 h (Beckman Coulter, Optima XPN-100), the exosomes were pelleted and stored at  80 °C for further use.

### Characterization of purified exosomes

The collected exosomes were suspended in PBS and stored at 4 °C in the Electron Microscopy Room at the Pathology Research Center of Xiangya Hospital for verification by electron microscopy.

The concentration and size of the purified exosomes were detected by NanoSight NS300 (Malvern Instruments), which is built in with a fast video-capturing and particle-tracking application.

Quantification of protein concentration in exosomal fractions was performed using the BCA protein assay kit as per the manufacturer's instructions(NCM Biotech).

### Internalization of exosomes by BMDMs

To examine the purified exosomes by fluorescence microscopy, the exosomes were labeled with PKH26 (Sigma) and centrifuged at 100,000 × g at 4 °C for 2 h after washing with PBS. Then, the labeled exosomes were cultured with BMDMs for 6 h at a final concentration of 20 μg/ml. Further, the cells were fixed by 4% paraformaldehyde for 20 min and stained with DAPI for 20 min. After washing with PBS, the cells were finally investigated and photographed using a confocal imaging system (Olympus FV1200).

### Animals

The BALB/C mice, C57BL/6 mice and SD rats (all male) were acquired from the Experimental Animal Center of The Third Xiangya Hospital of Central South University. We used 4- to 6-week-old mice and 3- to 4-week-old rats for all experiments. The animals were maintained in plastic cages at 60% relative humidity and 21 ± 2 °C under pathogen-free conditions. Lighting was provided based on a 12 h light-12 h dark cycle. All mice and rats were fed in pathogen-free facilities at The Third Xiangya Hospital of Central South University. All experimental procedures involving the mice and rats had been approved by the Animal Care and Use Ethics Committee of Central South University, and the best attempt was made to reduce the use of animals and their suffering throughout the experiments.

### In vivo mouse study

To establish the allotransplantation model with C57BL/6 mice, skin allografts were transplanted as previously described [67]. BALB/C skin-removed fascia (1 cm × 1.5 cm) were transplanted into C57BL/6 mice, and rapamycin (1 mg/kg) was injected into the recipient mice on the first three days after transplantation. Tail vein injections of exosomes (50 mg/kg) were performed once in 3 days for a total of 3 times. If the necrosis in the donor skin tissue was over 80%, it indicated the occurrence of rejection [41]. Then, hematoxylin & eosin (H&E) staining was conducted on the paraffin sections of skin grafts. The mice were euthanized properly before taking skin and spleen samples. Allocation of mice was implemented on a random basis. Downstream analysis of mouse samples (immunohistochemistry and flow cytometry) was performed in a blinded manner.

### Western blotting

To perform Western blotting, cells were collected, lysed in the RIPA buffer containing PMSF (100 mmol/ml), and then sonicated properly. The protein concentrations were detected by the BCA protein assay kit (NCM Biotech). The exosomal proteins were separated by 12% SDS–PAGE. The cell lysates were separated by 12% or 8% SDS–PAGE, and were then shifted onto nitrocellulose membranes. The blots were blocked by 5% nonfat dry milk for 90 min at room temperature and incubated with different primary antibodies at dilutions as instructed by the manufacturers at 4 °C overnight. Thereafter, HRP-conjugated secondary antibodies (Cell Signaling Technology) were added and the samples were incubated for 1 h at room temperature. ECL detection reagents (Bio-Sharp) were used to develop the blots. In BMDM, the protein expression of NLRP3 (Abcam, ab263899, 40 μl), NF-κB (Abcam, ab32536, 40 μl), procaspase-1 (Abcam, ab179515, 40 μl), and ASC (Cell Signaling Technology, 67824 T, 20 μl) was detected. CD63, CD9, TSG101, and HSP90B1 were used as exosome markers [68, 69], and β-actin was chosen as the loading control. At least three parallel tests were carried out to confirm the final results.

### Flow cytometry

Single-cell suspensions of the cultured BMDMs and splenocytes were prepared. Cell surface staining was then performed using AMCYAN-Live, PE-Cy7-CD45, PE-CD11b, BV421-F4/80, and APC-CD86 antibodies (Invitrogen) on ice in a dark environment for 30 min. For intracellular cytokine staining, the cells were first placed on ice for 45 min using a fixation/permeabilization kit, and then were incubated with FITC-CD206 antibodies (Invitrogen) on ice in a dark environment for 1 h after proper washing with the Perm/Wash buffer twice. Finally, all samples were washed with the Perm/Wash buffer twice to proceed with flow cytometry and FlowJo analyses.

### ELISA

ELISA plates (96-well) (eBioscience) were used to detect cytokines in the culture supernatant. After centrifugation at 3000 rpm at room temperature for 20 min, the BMDM culture supernatant was collected from each group. Then, 50 μl of supernatant was added to the plates coated with monoclonal antibodies, and the cultures were incubated at room temperature for 90 min. Subsequently, the wells were washed with wash buffer for three times, and any residual buffer was removed. Next, 100 μl of horseradish peroxidase-conjugated streptavidin (BD Biosciences) which was diluted with PBS was added to the plates, and the cultures were incubated at 37 °C for 1 h. After washing with wash buffer for five times, 50 μl of developer A and 50 µl of developer B were added sequentially to the plates, and the cultures were further incubated at 37 °C in a dark environment for 30 min. Lastly, 50 μl of stop solution was added to the plates, and the absorbance was read at 450 nm. The data were then collected and analyzed.

### Histological analysis

The mice were sacrificed 7 d after transplantation. The transplanted skin samples were excised and fixed in 4% formalin (v/v) at 4 °C overnight, then dehydrated in 30%, 50%, 70%, 80%, 90% and 100% ethanol for 1 h each after washing with PBS twice, and soaked in xylene for 4 h. Subsequently, the samples were embedded in paraffin and were cut into 5 μm sections for histological staining. H&E staining was employed to determine the severity (i.e., 0, normal; 1, mild; 2, modest; and 3, severe) of inflammatory cell infiltration in a blinded manner [26].

### Immunohistochemical staining

For immunohistochemical staining, the skin sections were heat-fixed at 60 °C for 2 h, deparaffinized in xylene for 30 min, and then rehydrated in 100%, 95%, 90%, 80%, and 70% ethanol for 10 min. After washing with PBS and PBST (PBS containing 0.05% Tween-20) for 5 min each, the sections were incubated with the endogenous peroxidase blocking buffer (Beyotime Biotechnology; P0100A, 100 ml) for 10 min. Next, the sections were washed with PBS for three times, and antigen retrieval was conducted for 10 min by steaming in 10 mM citrate antigen retrieval solution. Further, the sections were blocked with 3% bovine serum albumin (BSA; Sigma, V900933, 100 g) which was diluted by PBS for 1 h, then stained with F4/80, CD86, and CD206 at 4 °C overnight, and washed with PBST and PBS properly. The stained sections were incubated in a hypersensitive HRP-labeled goat anti-mouse/rabbit Ig mixture for 20 min and incubated with the signal enhancer reagent. After washing with PBST and PBS, DAB reagent was added and the sections were further incubated for 120 s. Thereafter, the samples were re-stained with hematoxylin for 60 s. Finally, the sections were dehydrated with 70%, 80%, 90%, 95%, 100% ethanol for 1 min each, with xylene for 10 min, and covered with neutral balsam for imaging and observation under a Leica fluorescence optical microscope.

### Immunofluorescence staining

For immunofluorescence staining, antigen retrieval was performed by boiling 0.1 M citrate buffer (pH = 6.0) for 10 min after dewaxing with xylene and rehydrating the samples. After being washed with PBS, permeabilization with 0.3% Triton X-100 (Invitrogen™, 10 ml, HFH10) was performed on all sections for 15 min before blocking by 5% bovine serum albumin (BSA, Sigma-Aldrich, 1 kg, V900933) for 1 h. The paraffin-embedded sections were incubated overnight at 4 °C with primary antibodies. Following this, the sections were washed with PBS and incubated with fluorophore-conjugated secondary antibodies at room temperature for 1 h, ensuring avoidance of exposure to light. Finally, the samples were stained with DAPI (Thermo Scientific™, 1 ml, 62,248) for 20 min, washed with PBS, and observed using a Leica confocal microscope.

### MiRNA microarray assay

The miRNA array analysis was performed by Guangzhou RiboBio Co., Ltd (Guangzhou, China) using exosome and LPS-pre Exos samples. The isolated RNA was assessed using the K5500 and Agilent 2200 TapeStation (Agilent Technologies, USA). Subsequently, the purified RNA underwent RT-PCR amplification and size selection using PAGE gel following the instructions of the NEBNext® Multiplex Small RNA Library Prep Set to generate Illumina®-compatible libraries of appropriate size. The quality of the purified library products was evaluated using the Agilent 2200 TapeStation. Finally, the libraries were subjected to single-end 50 bp sequencing on the HiSeq 2500 platform (Illumina, USA) at Ribobio Co. Ltd (Ribobio, China). For further pathway analysis, Gene Ontology (GO) and KEGG (Kyoto Encyclopedia of Genes and Genomes) databases were employed. GO and KEGG analyses for differentially expressed miRNAs identified downstream genes regulated by miRNAs (*P* < 0.001, |log2 (fold change)|> 1.8).

### Real-time RT-PCR

Total RNA from exosomes and LPS-pre Exos was isolated by using Total RNA Purification Micro Kit (Norgen, Biotek Corp.,Canada) according to the protocol of manual. We used NanoDrop 2000 spectrophotometer (NanoDrop Technologies, Thermo Scientific, USA) to detect the purity and content of total RNA. MiRNA 1st Strand cDNA Synthesis Kit (Accurate Biotechnology, Hunan, China) was used to synthesize cDNA. The SYBR^®^ Green Premix II Pro Taq HS qPCR Kit reagent (Accurate Biotechnology, Hunan, China) was employed as per the manufacturer's instructions for quantitative RT-PCR analysis. The QuantStudio 5 real-time PCR system (Thermo Fisher) was utilized for this purpose. The expression level of the target gene was determined using the 2-ΔΔCt method, wherein the Ct value of U6 was subtracted to calculate the relative expression level. The primers used in the real-time RT-PCR experiments were purchased from Accurate Biotechnology (Hunan, China) and are listed in Table 1.

**Table1:** The miRNA primer sequences used in PCR detection

The name of MiRNAs	Primer (5’ to 3’)	Primer sequence
MiR-486	ForwardReverse	GCAGTCCTGTACTGAGCTG
MiR-206-3p	ForwardReverse	GCAGTGGAATGTAAGGAAGT
MiR-409a-3p	ForwardReverse	GCAGAATGTTGCTCGGT
MiR-543-3p	ForwardReverse	GCAGAAACATTCGCGGTG
MiR-144	ForwardReverse	GCGCAGGGATATCATCATATAC
MiR-222-3p	ForwardReverse	GCAGAGCTACATCTGGCT
MiR-1b	ForwardReverse	CGCAGTGGAATGTAAAGAAG
MiR-411-3p	ForwardReverse	CGCAGTATGTAACACGGT

### Statistical analysis

All tests were conducted for three times at least, including both in vitro and in vivo (5 animals in each group). The results were expressed as the mean ± standard deviation or SEM. GraphPad Prism 9.0 was used for statistical analysis. The significance of mean differences was determined by the Student’s t test or one-way analysis of variance to evaluate the effect of exosome treatment. P < 0.05 was considered statistically significant.

### Supplementary Information

**Additional file 1: Fig. S1 (A)** Representative photomicrographs of BM-MSCs and LPS pre-MSCs in culture showing similar fusiform and triangular morphologies. Scale bar 100μm. **(B)** LPS pre-MSCs differentiated into osteocytes, chondrocytes and adipocytes. **(C)** Characteristic cell surface markers of LPS pre-MSCs were detected by flow cytometry. **(D)**, **(E)** After appropriate lipopolysaccharide stimulation, Live-Amcyan staining flow analysis showed no significant difference in the mortality of MSCs between the two groups (n = 3). Data are presented as mean ± SD. ns P>0.05. **Fig. S2 **The markers (HSP90B1, CD63, TSG101, CD9) of exosomes analyzed by western blot. Full-length blots are presented in Supplementary Figure 2. Blots: Origin images of Western blots; White: Origin images of transferred PVDF membranes; Merge: The origin images of which blots merged with the PVDF membrane. The blots marked in red are the parts cropped. **Fig. S**3 BMDM cells were pretreated with LPS (100ng/m) for 3h before culturing with LPS pre-Exo for 24h. Protein expression of NF-κB, NLRP3, Procaspase-1 and ASC in macrophages of each group was analyzed by western blot. Full-length blots are presented in Supplementary Figure 2. Blots: Origin images of Western blots; White: Origin images of transferred PVDF membranes; Merge: The origin images of which blots merged with the PVDF membrane. The blots marked in red are the parts cropped. **Fig. S4 **BMDM cells were pretreated with LPS (100ng/m) for 3h before culturing with LPS pre-Exo for 48h. Protein expression of NF-κB, NLRP3, Procaspase-1 and ASC in macrophages of each group was analyzed by western blot. Blots: Origin images of Western blots; White: Origin images of transferred PVDF membranes; Merge: The origin images of which blots merged with the PVDF membrane. The blots marked in red are the parts cropped.

## Data Availability

All relevant data and materials are available from the authors upon reasonable request.

## Abbreviations

LPS: Lipopolysaccharide
MSCs: Mesenchymal stem cells
LPS pre-MSCs: LPS-pretreated MSCs
LPS pre-Exos: LPS-pretreated MSC-derived exosomes
BMDMs: Bone marrow-derived macrophages
BM-Exos: MSC-derived exosomes
Bm-MSCs: Bone marrow-derived MSCs
NF-κΒ: Nuclear factor kappa B
NLRP3: NOD-like receptor thermal protein domain associated protein 3
GvHD: Graft-versus-host disease
LPS pre-BM-MSCs: LPS-preconditioned bone marrow MSCs
ASC: Apoptosis-associated speck-like protein
MEM-α: Minimum essential medium-alpha
FBS: Fetal bovine serum
PBS: Phosphate buffered solution
DMEM: Dulbecco's modified Eagle’s medium
M-CSF: Macrophage-stimulating factor
PMSF: Phenylmethanesulfonyl fluoride
DAPI: 4′,6-Diamidino-2-phenylindole
PBST: PBS with 0.05% Tween-20
NTA: Nanoparticle tracking analysis
TEM: Transmission electron microscopy
POD: Postoperative day
PAMPs: Pathogen-associated molecular pattern
DAMPs: Damage associated molecular patterns
ATP: Adenosine-triphosphate
ER: Endoplasmic reticulum
