# Supplementary material for: Exosomes derived from LPS-preconditioned bone marrow-derived MSC modulate macrophage plasticity to promote allograft survival via the NF-κB/NLRP3 signaling pathway
Source: J Nanobiotechnology. 2023 Sep 16;21:332. doi: 10.1186/s12951-023-02087-8 (PMC10504750; doi:10.1186/s12951-023-02087-8)
Supplement: Supplementary file 1 — Additional file 1: Fig. S1 (A) Representative photomicrographs of BM-MSCs and LPS pre-MSCs in culture showing similar fusiform and triangular morphologies. Scale bar 100μm. (B) LPS pre-MSCs differentiated into osteocytes, chondrocytes and adipocytes. (C) Characteristic cell surface markers of LPS pre-MSCs were detected by flow cytometry. (D), (E) After appropriate lipopolysaccharide stimulation, Live-Amcyan staining flow analysis showed no significant difference in the mortality of MSCs between the two groups (n = 3). Data are presented as mean ± SD. ns P>0.05. Fig. S2 The markers (HSP90B1, CD63, TSG101, CD9) of exosomes analyzed by western blot. Full-length blots are presented in Supplementary Figure 2. Blots: Origin images of Western blots; White: Origin images of transferred PVDF membranes; Merge: The origin images of which blots merged with the PVDF membrane. The blots marked in red are the parts cropped. Fig. S3 BMDM cells were pretreated with LPS (100ng/m) for 3h before culturing with LPS pre-Exo for 24h. Protein expression of NF-κB, NLRP3, Procaspase-1 and ASC in macrophages of each group was analyzed by western blot. Full-length blots are presented in Supplementary Figure 2. Blots: Origin images of Western blots; White: Origin images of transferred PVDF membranes; Merge: The origin images of which blots merged with the PVDF membrane. The blots marked in red are the parts cropped. Fig. S4 BMDM cells were pretreated with LPS (100ng/m) for 3h before culturing with LPS pre-Exo for 48h. Protein expression of NF-κB, NLRP3, Procaspase-1 and ASC in macrophages of each group was analyzed by western blot. Blots: Origin images of Western blots; White: Origin images of transferred PVDF membranes; Merge: The origin images of which blots merged with the PVDF membrane. The blots marked in red are the parts cropped. [file 12951_2023_2087_MOESM1_ESM.docx]

Supplementary Material

**Supplementary Figures**


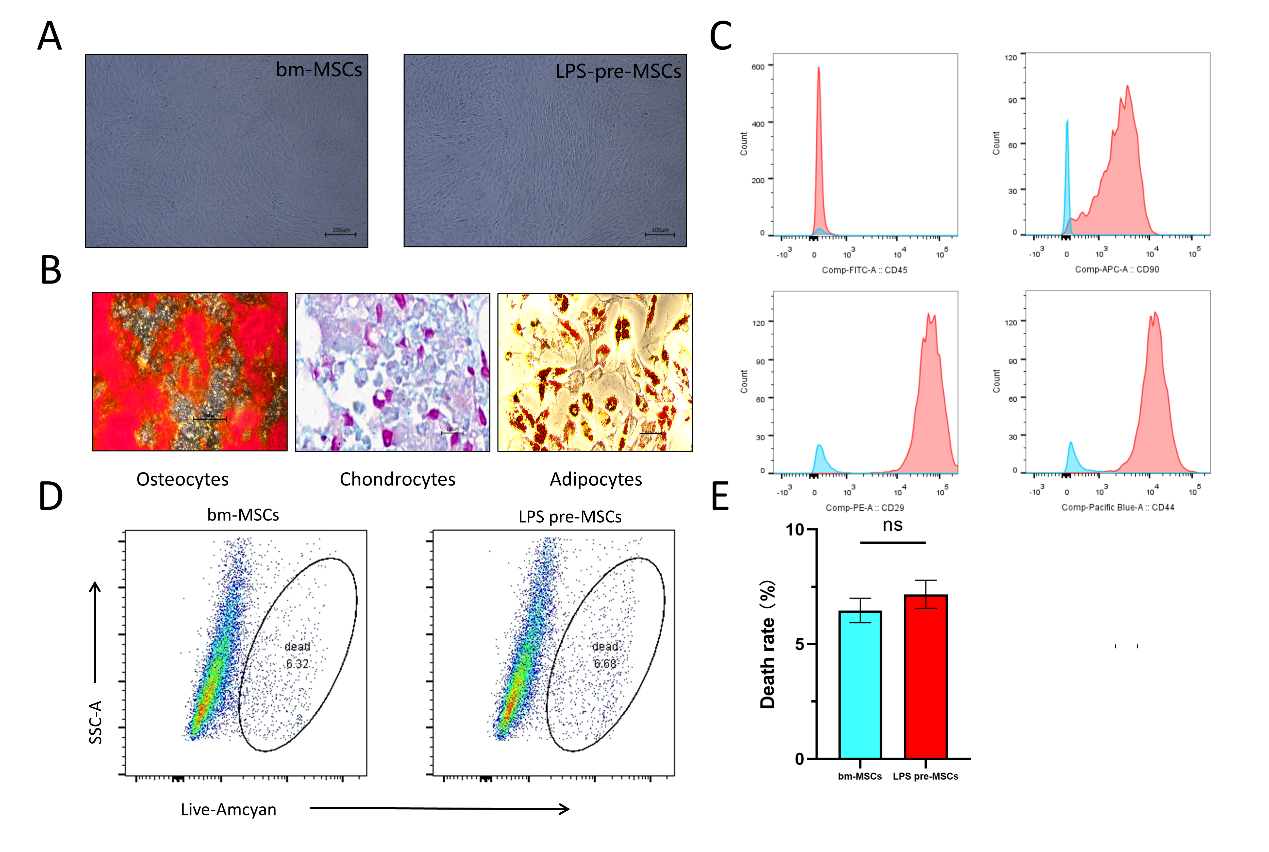


**Supplementary Fi.1 (A)** Representative photomicrographs of BM-MSCs and LPS pre-MSCs in culture showing similar fusiform and triangular morphologies. Scale bar 100μm. **(B)** LPS pre-MSCs differentiated into osteocytes, chondrocytes and adipocytes. **(C)** Characteristic cell surface markers of LPS pre-MSCs were detected by flow cytometry. **(D)**, **(E)** After appropriate lipopolysaccharide stimulation, Live-Amcyan staining flow analysis showed no significant difference in the mortality of MSCs between the two groups (n = 3). Data are presented as mean ± SD. ns P>0.05


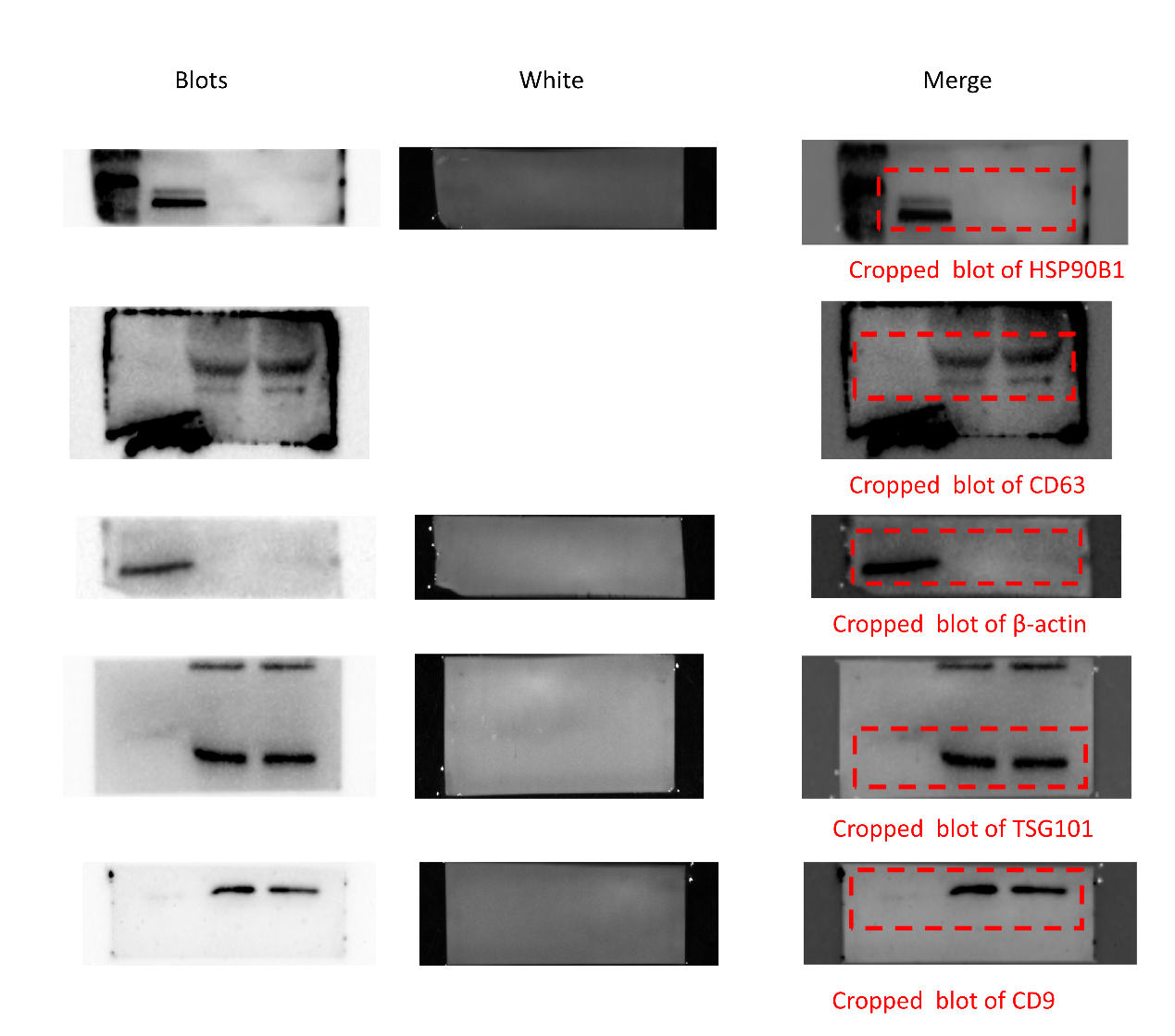


**Supplementary Fig.2** The markers (HSP90B1, CD63, TSG101, CD9) of exosomes analyzed by western blot. Full-length blots are presented in Supplementary Figure 2. Blots: Origin images of Western blots; White: Origin images of transferred PVDF membranes; Merge: The origin images of which blots merged with the PVDF membrane. The blots marked in red are the parts cropped.


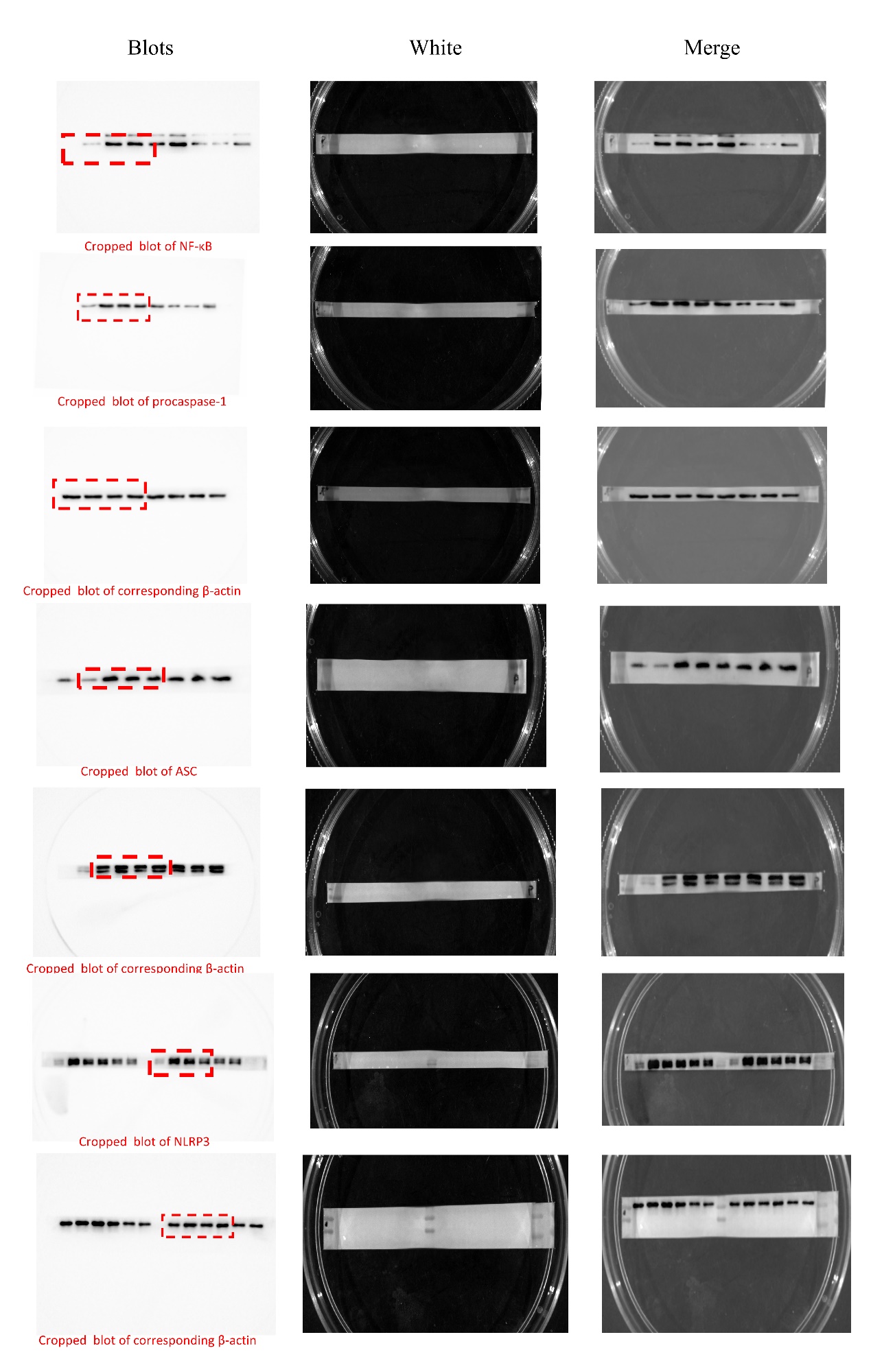


**Supplementary Fi.** 3 BMDM cells were pretreated with LPS (100ng/m) for 3h before culturing with LPS pre-Exo for 24h. Protein expression of NF-κB, NLRP3, Procaspase-1 and ASC in macrophages of each group was analyzed by western blot. Full-length blots are presented in Supplementary Figure 2. Blots: Origin images of Western blots; White: Origin images of transferred PVDF membranes; Merge: The origin images of which blots merged with the PVDF membrane. The blots marked in red are the parts cropped.


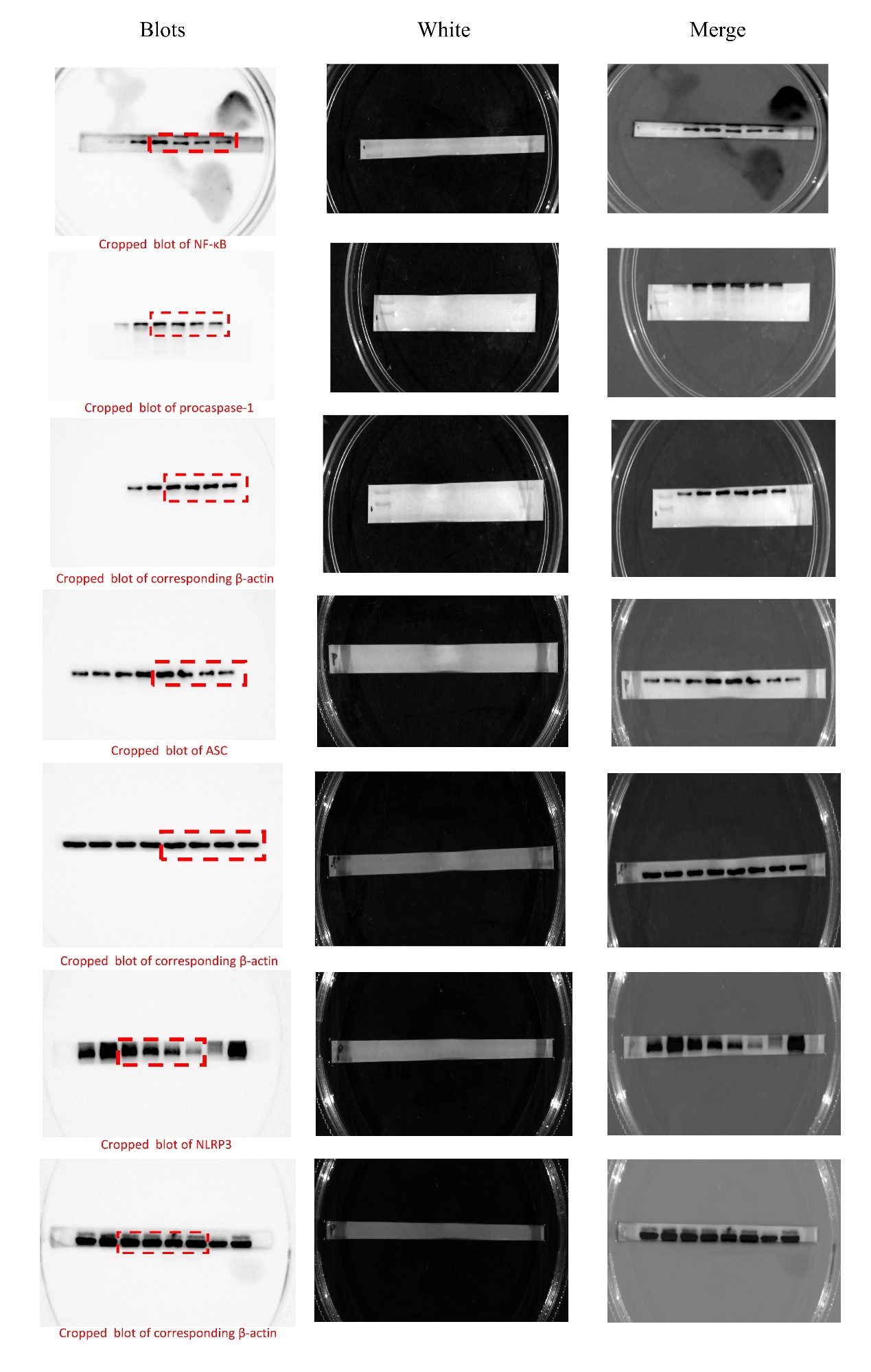


**Supplementary Fig.4** BMDM cells were pretreated with LPS (100ng/m) for 3h before culturing with LPS pre-Exo for 48h. Protein expression of NF-κB, NLRP3, Procaspase-1 and ASC in macrophages of each group was analyzed by western blot. Blots: Origin images of Western blots; White: Origin images of transferred PVDF membranes; Merge: The origin images of which blots merged with the PVDF membrane. The blots marked in red are the parts cropped.
